# Gender-based violence among people with disabilities is a neglected public health topic

**DOI:** 10.1080/16549716.2019.1694758

**Published:** 2019-11-28

**Authors:** Fredinah Namatovu, Raman Preet, Isabel Goicolea

**Affiliations:** a Centre for Demographic and Ageing Research (CEDAR), Umeå University, Umeå, Sweden; b Department of Epidemiology and Global Health, Faculty of Medicine, Umeå University, Umeå, Sweden

**Keywords:** Gender and Health Inequality, Gender-based violence, disability, service provision

## Abstract

This paper aims to provide an analytical insight on the current state of knowledge on gender-based violence among people with disabilities, a topic where the level of data is relatively low. We briefly discuss the current research on: (a) the prevalence, risk factors and the theoretical approaches for gender-based violence among people with disabilities. (b) Service provision among people with disabilities who experience gender-based violence. (c) We also highlight areas where further research is required, the applicable theoretical approaches and provide an example on how Sweden is attempting to bridge this knowledge gap through implementing the Disability and Intimate-partner violence project (DIS-IPV) project.

## Background

Gender-based violence (GBV) defined, as any physical, sexual and psychological harm remains a major public health problem despite numerous written political commitments, policies and programmes [1,2]. Social discriminations such as those based on gender and disability increase the vulnerability to GBV for both men and women with disabilities [3]. Even though both men and women with disabilities experience GBV, women and girls with disabilities are at an increased risk [3]. The social model defines disability as a social construct that arises as society disables people with impairments by denying them full participation [4]. To-date there is scanty research on the prevalence and risk factors of GBV among people with disabilities (PWDs) [5–8]. Moreover, research evaluating services and interventions that address GBV among PWDs is almost inexistent [9]. This eminent lack of data concerning PWDs obscures the state of GBV in this population, deterring political commitment and comprehensive public health engagement. In this paper, we aim to present the existing landscape of research on GBV with a specific focus on GBV-related services for PWDs, and exemplify a recently implemented project, in Sweden that seeks to address this research gap.

### GBV among people with disabilities

The few existing studies on GBV among PWDs indicate that PWDs are at equal or greater risk of GBV compared to their peers without disabilities [5–14]. PWDs report multiple forms of violence during their lifetime, by multiple perpetrators and for longer periods, compared to people without disabilities [5]. PWDs are vulnerable to violence from family members and well-known acquaintances just like people without disabilities [6,8,9]. Existing research rarely considers gender differences in investigating GBV among PWDs yet those that do, report conflicting results. Some studies indicate that men and women with disabilities are at equal or greater risk of GBV compared to their peers without disabilities [5]. Others suggest that women with disabilities have higher rates of emotional, sexual and physical abuse compared to women without disabilities [6,11]. Some studies indicate that more women than men with disabilities are likely to identify an intimate partner as their abuser, whereas, men are more likely to report disability service providers as their abusers [3]. Some data reveal higher rates of interpersonal violence among men with disabilities compared to either women or men without disabilities [9].

Looking at Sweden, GBV research among PWDs is still limited here as well. The existing data suggest that the nature of violence experienced differs by type of disability and gender [10]. Men with physical disabilities report a greater risk of physical and psychological violence compared to men without disabilities, whereas women with auditory impairments are at increased risk of physical and psychological violence [10]. Women with disabilities report being afraid of subjection to violence, to tolerate abuse and are less likely to report the abuse [15]. Women with disabilities in need of assistance in their everyday lives report increased vulnerability to GBV linked to dependence on other people in their surroundings [15]. Perpetrators of violence are both men and women, including family members and service providers [13,15].

The health consequences of GBV among PWDs are yet another understudied area both globally and in Sweden. Intimate partner violence is associated with negative mental and physical health; effects include chronic gastrointestinal, gynaecologic, cardiovascular and mental health issues [2,16]. Correspondingly, research on the health consequences of GBV among PWDs is limited but existing data suggests a link between abuse and poor health outcomes among PWDs [14].

### Determinants of gender-based violence among people with disabilities

Factors that increase the vulnerability of PWDs are multifaceted. Gender role attitudes are one of the key determinants that increase GBV vulnerability among women with disabilities [17,18]. Several societies control the sexuality of women with disabilities through practices such as institutionalisation, forced sterilization and marriage restriction [18]. Additionally, women with disabilities are socialised to be agreeable in order to receive care. Such socialisation creates internalised oppression and compliance hindering them from reporting abuse and encouraging them to remain in abusive situations [19]. Such social norms that promote societal devaluation of women with disabilities increase their vulnerability to GBV [19].

The social context of disability is yet another key exposure to GBV among PWDs. Disabilities that require dependency on others for support are exploited by perpetrators to exercise power and control, which increases vulnerability to GBV [19]. A study in the US show that women with functional limitations and in need of assistance with daily activities report emotional, sexual and physical abuse from family and non-family paid workers [6]. The risk to violence also appears to differ by type of disability [5,15]. People with mental disabilities are at increased risk to GBV because perpetrators regard them as ‘easy targets’. Although PWDs were 1.5 times more likely to be victims of violence than those without disabilities, those with disabilities associated with mental illnesses were at nearly four times higher risk of experiencing violence [5].

Structural barriers increase the vulnerability to GBV among PWDs [6,18]. Such structural barriers include lack of access to resources and support systems, lack of political attention on GBV in PWDs, inadequate training for service providers and lack of knowledge on existing services and inaccessibility of services by PWDs [18–21].

### Theoretical approaches

We identified two theoretical approaches that can prove useful in understanding GBV among PWDs. The first approach is using the intersectionality approach that views social identities such as gender, ability, race and sexuality as interdependent and interconnected [22]. Such identities create different experiences based on population diversity and thus, should not be studied independent of one another nor in separation from prevailing societal processes [22–24]. Several factors that increase the risk of GBV among men and women with disabilities are interlinked; the intersectionality approach appears useful in studying GBV among PWDs through examining all the multifaceted factors.

The second approach combines the social model of disability with the material feminism [24]. The social model views disability as a social construct, rather than an impairment. This model suggests that disability in itself does not affect one’s participation but rather the prevailing social conditions deny PWDs full societal participation [24]. Material feminism advances the social model adding that existing historical, social and economic conditions institutionalised by the patriarchal societies create privileges for men giving them power over women; these privileges are then used to control and abuse women [25]. Combining the social model of disability and the material feminism is useful in understanding the increased GBV risk among women with disabilities.

## Services for people with disabilities experiencing gender-based violence

Evidence indicates that GBV-related services offered to survivors by professional service providers can reduce the risk to severe and fatal violence [26], and that service providers’ response is a key remedial factor in prevention of GBV recurrence among survivors [27]. Access to quality social support reduces the adverse consequences of GBV on women leading to improved health outcomes [7,28–30]. However, literature shows absence of studies assessing access to support services for women with disabilities experiencing GBV [21]. The little available literature reveals difficulties in accessing GBV-related services, especially among people with visual and hearing impairments [19,21]. Additionally, there is scant research on the competence of various service providers working with PWDs who experience GBV.

### Service provision for people with disabilities in Sweden

In Sweden, people exposed to GBV seeking professional help mainly turn to health care, to the social services and to non-governmental women’s shelters [31]. Sweden’s social services and the health-care system aim at upholding public commitment to equal access to services and thus to reducing inequalities between social groups [32]. The availability of different GBV services should make Sweden a relatively low threshold country for help seeking among PWDs exposed to GBV. However, to our knowledge no study has taken a holistic approach to evaluate the availability, access and efficacy of GBV-related services and interventions for PWDs. Consequently, it is unclear whether such services adequately meet the needs of PWDs.

To address this research gap, we are currently implementing “the Disability and Intimate Partner Violence (DIS-IPV)“ project in Sweden. We focus on intimate partner violence (IPV) because it is one of the frequently reported forms of GBV among PWDs. The aim of this project is to advance knowledge on the needs of PWDs exposed to IPV by assessing how existing services address their needs. The findings of this project will help to strengthen access and improve the quality of IPV services in this population. This project reaches out to PWDs exposed to IPV and to service providers, including health-care professionals, police, social workers and staff at shelters responsible for delivering GBV-related services. Our study uses a mixed methods approach of in-depth interviews and a cross-sectional survey for data collection. We use the intersectionality framework to understand how factors related to gender and disability influence access to IPV-related services. Combining the social model and the material feminist framework is ideal in identifying gender inequalities in service provision that affect adequate access to services among women with disabilities. We anticipate a rich collection of experiences that we will analyse and share with the public health services and scholars in Sweden and elsewhere.

## Conclusion

GBV is rooted in gender inequalities and thus research on GBV among PWDs requires application of frameworks that consider the dimensions of gender and disability. Relevant analytical frameworks should put into account the gender power relations that create subordination of people with disabilities. The intersectionality framework allows underpinning the complex multidimensional factors that expose women PWDs to GBV. In addition, combining the social model and the material feminist framework is ideal as it clearly illustrates that all women whether with disability or not have vulnerabilities that are used by perpetrators to exert power and control; however, disability adds an additional layer of vulnerability. The identified research gaps include a lack of data on GBV prevalence, risk factors and consequences. Existing research on GBV does not fully encompass all PWDs as it lacks disaggregation by gender, type of disabilities and other social categories such as ethnicity-racialization, sexual identity orientation or expression. We identified a critical need to evaluate how existing services address the needs of PWDs exposed to GBV because this evidence is almost non-existent. Future research on all aspects of GBV among PWDs will provide policy-relevant empirical evidence valuable in fostering political commitment and subsequent comprehensive public health engagement.

## Paper context

The number of people with disabilities is increasing globally due to ageing and medical advancement yet there is limited research focusing on the different aspects of gender-based violence in this population. This paper provides the current research scope on gender-based violence among people with disabilities with special focus on service provision, a neglected research topic. The authors highlight areas requiring further research attention and provide an example of how Sweden is addressing this research gap.

## References

[1] Garcia-Moreno C, Jansen HA, Ellsberg M, et al. Prevalence of intimate partner violence: findings from the WHO multi-country study on women’s health and domestic violence. Lancet. 2006;368:97–100. PubMed PMID: 17027732. 10.1016/S0140-6736(06)69523-8 17027732

[2] Krug EG, Mercy JA, Dahlberg LL, et al. The world report on violence and health. Lancet. 2002;360:1083–1088. PubMed PMID: 12384003. 12384003 10.1016/S0140-6736(02)11133-0

[3] Platt L, Powers L, Leotti S, et al. The role of gender in violence experienced by adults with developmental disabilities. J Interpers Violence. 2015;32:101–129. 25979536 10.1177/0886260515585534

[4] Davis LJ. The disability studies reader. London: Routledge; 1997.

[5] Hughes K, Bellis MA, Jones L, et al. Prevalence and risk of violence against adults with disabilities: a systematic review and meta-analysis of observational studies. Lancet. 2012;379:1621–1629. PubMed PMID: WOS:000303452600036. 22377290 10.1016/S0140-6736(11)61851-5

[6] Nosek MA, Foley CC, Hughes RB, et al. Vulnerabilities for abuse among women with disabilities. Sex Disabil. 2001;19:177–189.

[7] Postmus JL, Severson M, Berry M, et al. Women’s experiences of violence and seeking help. Violence against Wom. 2009;15:852–868. PubMed PMID: 19458091. 10.1177/1077801209334445 19458091

[8] Cohen MM, Forte T, Du Mont J, et al. Adding insult to injury: intimate partner violence among women and men reporting activity limitations. Ann Epidemiol. 2006;16:644–651. PubMed PMID: 16516492. 16516492 10.1016/j.annepidem.2005.12.005

[9] Ballan MS, Freyer MB, Powledge L. Intimate partner violence among men with disabilities: the role of health care providers. Am J Men’s Health. 2017;11:1436–1443. PubMed PMID: 26400712. 26400712 10.1177/1557988315606966 PMC5675191

[10] Olofsson N, Lindqvist K, Danielsson I. Higher risk of violence exposure in men and women with physical or sensory disabilities: results from a public health survey. J Interpers Violence. 2015;30:1671–1686. PubMed PMID: 25186966. 25186966 10.1177/0886260514548585

[11] del Rio Ferres E, Megias JL, Exposito F. Gender-based violence against women with visual and physical disabilities. Psicothema. 2013 Feb;25:67–72. PubMed PMID: 23336546. 23336546 10.7334/psicothema2012.83

[12] Krnjacki L, Emerson E, Llewellyn G, et al. Prevalence and risk of violence against people with and without disabilities: findings from an Australian population-based study. Aust N Z J Public Health. 2016 Feb;40:16–21. PubMed PMID: 26714039. 26714039 10.1111/1753-6405.12498

[13] Zijlstra E, Esselink G, Moors ML, et al. Vulnerability and revictimization: victim characteristics in a dutch assault center. J Forensic Leg Med. 2017;52:199–207. 28961551 10.1016/j.jflm.2017.08.003

[14] Hughes RB, Robinson-Whelen S, Raymaker D, et al. The relation of abuse to physical and psychological health in adults with developmental disabilities. Disabil Health J. 2019;12:227–234. 30655190 10.1016/j.dhjo.2018.09.007

[15] Handu AB Swedish research institute for disability policy. Level of living survey 2005. [cited 2019 May 22]. Available from: http://www.handu.se/research-institute-handu-ab/2007

[16] Campbell JC. Health consequences of intimate partner violence. Lancet. 2002;359:1331–1336. PubMed PMID: 11965295. 11965295 10.1016/S0140-6736(02)08336-8

[17] Sobsey R. Violence and abuse in the lives of people with disabilities: the end of silent acceptance? Baltimore (MD): Paul H Brookes Publishing; 1994.

[18] Chenoweth L. Violence and women with disabilities: silence and paradox. Violence against Women. 1996;2:391–411.

[19] Saxton M, Curry MA, Powers LE, et al. Bring my scooter so i can leave you”: a study of disabled women handling abuse by personal assistance providers. Violence against Wom. 2001;7:393–417.

[20] Swedlund NP, Nosek MA. An exploratory study on the work of independent living centers to address abuse of women with disabilities. J Rehabil. 2000;66:57–64. PubMed PMID: 3904326.

[21] Mandl S, Schachner ACS, Planitzer J. Access to Specialised Victim Support Services for Women with Disabilities who have experienced Violence. Ludwig Boltzmann Institute of Human Rights (BIM) and queraum; 2014. [cited 2019 June 19]. Available from: http://fotlunarfraedi.hi.is/access_to_specialised_victim_support_services_for_women_with_disabilities_who_have_experienced

[22] Shaw LR, Chan F, McMahon BT. Intersectionality and disability harassment: the interactive effects of disability, race, age, and gender. Rehabil Couns Bull. 2011;55:82–91.

[23] Sommo A, Chaskes J. Intersectionality and the disability: some conceptual and methodological challenges. In: Barnartt SN, Altman BM, editors. Disability and intersecting statuses. Vol. 7. Research in Social Science and Disability. Emerald Group Publishing Limited; 2013;7:47–59.

[24] Hamilton R. The liberation of women (RLE Feminist Theory): A study of patriarchy and capitalism. London, United Kingdom: Routledge; 2012.

[25] Mays JM. Feminist disability theory: domestic violence against women with a disability. Disabil Soc. 2006;21:147–158.

[26] Ellsberg M, Heise L, Peña R, et al. Researching domestic violence against women: methodological and ethical considerations. Stud Fam Plann. 2001;32:1–16. 11326453 10.1111/j.1728-4465.2001.00001.x

[27] Garcia-Moreno C. Dilemmas and opportunities for an appropriate health-service response to violence against women. Lancet. 2002;359:1509–1514. PubMed PMID: WOS:000175279100027. 11988263 10.1016/S0140-6736(02)08417-9

[28] Dufort M, Gumpert CH, Stenbacka M. Intimate partner violence and help-seeking – a cross-sectional study of women in Sweden [journal article]. BMC Public Health. 2013;13:866. 24053735 10.1186/1471-2458-13-866 PMC3852487

[29] Selic P, Pesjak K, Kersnik J. The prevalence of exposure to domestic violence and the factors associated with co-occurrence of psychological and physical violence exposure: a sample from primary care patients. BMC Public Health. 2011;11:621. 21816070 10.1186/1471-2458-11-621 PMC3160996

[30] Liebschutz J, Battaglia T, Finley E, et al. Disclosing intimate partner violence to health care clinicians - what a difference the setting makes: a qualitative study. Bmc Public Health. 2008;8:229–229. PubMed PMID: 18601725. DOI:10.1186/1471-2458-8-229 PMC2474863 18601725

[31] Antilla S, Ericson C, Glad J, et al. Book utfall och effekter av sociala metoder för kvinnor som utsatts för våld i nära relationer: en systematisk översikt. Utfall och effekter av sociala metoder för kvinnor som utsatts för våld i nära relationer: en systematisk översikt. Stockholm: Socialstyrelsen; 2006.

[32] Lundberg O, MÅ Y, Stjärne MK, et al. The role of welfare state principles and generosity in social policy programmes for public health: an international comparative study. Lancet. 2008;372:1633–1640. 18994660 10.1016/S0140-6736(08)61686-4

